# A survey of visually induced symptoms and associated factors in spectators of three dimensional stereoscopic movies

**DOI:** 10.1186/1471-2458-12-779

**Published:** 2012-09-13

**Authors:** Angelo G Solimini, Alice Mannocci, Domitilla Di Thiene, Giuseppe La Torre

**Affiliations:** 1 Department of Public Health and Infectious Diseases, Sapienza University of Rome, Piazza Aldo Moro, 5, 00185, Rome, Italy

**Keywords:** Visually induced motion sickness, Cibersickness, Three dimensional (3D) movies

## Abstract

**Background:**

The increasing popularity of commercial movies showing three dimensional (3D) computer generated images has raised concern about image safety and possible side effects on population health.

This study aims to (1) quantify the occurrence of visually induced symptoms suffered by the spectators during and after viewing a commercial 3D movie and (2) to assess individual and environmental factors associated to those symptoms.

**Methods:**

A cross-sectional survey was carried out using a paper based, self administered questionnaire. The questionnaire includes individual and movie characteristics and selected visually induced symptoms (tired eyes, double vision, headache, dizziness, nausea and palpitations). Symptoms were queried at 3 different times: during, right after and after 2 hours from the movie.

**Results:**

We collected 953 questionnaires. In our sample, 539 (60.4%) individuals reported 1 or more symptoms during the movie, 392 (43.2%) right after and 139 (15.3%) at 2 hours from the movie. The most frequently reported symptoms were tired eyes (during the movie by 34.8%, right after by 24.0%, after 2 hours by 5.7% of individuals) and headache (during the movie by 13.7%, right after by 16.8%, after 2 hours by 8.3% of individuals). Individual history for frequent headache was associated with tired eyes (OR = 1.34, 95%CI = 1.01-1.79), double vision (OR = 1.96; 95%CI = 1.13-3.41), headache (OR = 2.09; 95%CI = 1.41-3.10) during the movie and of headache after the movie (OR = 1.64; 95%CI = 1.16-2.32). Individual susceptibility to car sickness, dizziness, anxiety level, movie show time, animation 3D movie were also associated to several other symptoms.

**Conclusions:**

The high occurrence of visually induced symptoms resulting from this survey suggests the need of raising public awareness on possible discomfort that susceptible individuals may suffer during and after the vision of 3D movies.

## Introduction

The worldwide increasing popularity of commercial movies showing stereoscopic (e.g. three dimensional; 3D) motion images is documented by the fact that 3D releases are generating more revenues than the same movie released in 2D[1]. In parallel with the expansion of digital 3D cinema systems, several consumer-electronics manufacturers released 3D televisions and displays for the home entertainment [2]. For example, more than 300 3D videogames are already available for computers and consoles [2]. Stereoscopic displays are becoming also very important for no leisure applications such as vision research, operation of remote devices, medical imaging, surgical training, scientific visualization, virtual prototyping, and many others [3]. In the near future, it is predictable that more and more people will pass increased portion of time (either leisure or work time) viewing 3D motion images, raising concern about the 3D image safety and possible adverse side effects on end users.

Stereoscopic display of motion images in movie theaters are not new (the first commercial movie was shown in a movie theater in Los Angeles in 1922 [4]) and many different techniques have been applied to obtain the stereoscopic illusion. With the advancement of digital technology, many drawbacks related to image generation, transmission, displays that could cause visual discomfort to viewers have been removed or tiled off. However, the strong sensorial exercise that is caused by the vision of a 3D movie can still evoke some adverse effects for health. It is known that the view of stereoscopic images imposes an intense eye accommodation activity, such as focusing and converging, that may result in visual fatigue [5] in some of the viewers. Depending on the specific field of investigation, those adverse effects have been referred as visual fatigue [6], asthenopia [7], eyestrain [8] or visually induced motion sickness [9] (VIMS). A further distinction has been made between objective and subjective conditions of visual fatigue. The latter has been termed visual discomfort and includes those symptoms indicative of (objective) physiological changes that decrease the performance of the visual system [6].

Symptoms of visual fatigue include eye discomfort and tiredness, pain and sore around the eyes, dry or watery eyes, headaches and visual distortions such as blurred and double visions, and difficult in focusing (see [5] for a full review). Large information on visual fatigue derives from studies dedicated to the computer vision syndrome suffered by video terminal operators (reviewed by Blehm and colleagues [10]).

VIMS is a condition in which viewers experience symptoms of visual fatigue nausea and dizziness during or after viewing dynamic images while being physically still [9], like people sitting in a movie theatre. Depending on the type of motion viewed by the stationary observers, VIMS has been referred to as cinerama sickness, flight simulator sickness, cibersickness (or virtual reality sickness) and others[11]. Some authors [12], [13] suggested that , although visual stress causing eyestrain and visually induced motion sickness are different conditions, they share some common mechanisms.

Information exists on adverse effects on viewers induced by virtual reality environments (see [14] and references therein), 3D displays (reviewed by [5] and [6]), simulators (reviewed by [9]), but little is known on the occurrence of symptoms when viewing a 3D movie in real life (e.g. non experimental) conditions. Previous experiences were limited in the number of subject enrolled by the experimental setups [8], [15] and the recent releases of blockbuster 3D movies have raised some concern on image safety and comfort for spectators.

This study aims to (1) quantify the occurrence of self reported symptoms suffered by the spectators during and after viewing a commercial 3D movie in cinemas and (2) to assess potential factors associated to those symptoms.

## Methods

### Study design and eligibility

A cross-sectional survey was designed to assess self reported symptoms, individual and 3D movie vision characteristics. A convenience sample was obtained during two distinct periods (15 March to 15 April 2010 and 15 December 2010 to 15 January 2011) from adult community members of coauthors and students of undergraduate and graduate public health courses (including continuing education courses) taught by two of us (GLT and AGS). Paper based, self administered questionnaires were distributed to those individuals declaring to have seen a 3D movie in a theater with dedicated 3D cinema room within the past 6 months. Exclusion criteria were as follow: not to have seen a commercial 3D movie in a dedicated cinema room, not remembering the movie title, not reporting the general health condition during the week of the movie vision at least as good.

### Ethic statement

The anonymous data collection procedures, in which subjects provided written informed consent, were approved by the Ethic Committee of the Sapienza University Hospital Policlinico Umberto I, Rome.

### Questionnaire

The questionnaire was designed with 23 items including individual and movie vision characteristics and selected VIMS symptoms. A previous questionnaire version with 20 items has been validated in a pilot study reported elsewhere[16]. Three additional items were added in this study to the original questionnaire to account for perceived anxiety level, movie show time and enjoyment and appreciation of the movie seen (see below). The questionnaire is anonymous.

### Measures

#### Individual characteristics

Individual characteristics include socio-demographic variables (gender, age, marital status, educational level, employment status), use of glasses or prescription lens when watching movies and possible individual predictors of symptoms as history for headache, frequency of car sickness, monthly frequency of vertigo disturbs (including other non specific forms of dizziness), self perceived anxiety level and daily exposure to the use of computer and/or video games (see Table 1 for modalities of variables). Headache, car sickness and dizziness are all possible correlates with individual sensitivity of the visual-vestibular system to external stimuli. Self perceived anxiety level was included to account for interaction between psychological and physiological processes in everyday situations. For example, anxiety level might be connected with increased symptom reporting in some individuals and has been reported as a trigger of migraine [17]. Use of computer and or videogames was measured because of the well known link between exposure to more than 4 hours at videoterminal and the increased risk of asthenopia [10], as visual fatigue can accumulate with prolonged visual stress[5].

**Table 1:** Socio-demographic characteristics of study participants

**Variable**		**N**	**(%)**
Age	18-29	612	67.5
	30-39	170	18.7
	40-49	93	10.3
	50-59	23	2.5
	> = 60	9	1.0
	Total	907	
Gender	Female	417	46.0
	Male	490	54.0
	Total	907	
Marital status	Married (or co-habitant with partner)	233	25.7
	Divorced	13	1.4
	Never married (nor currently cohabitant)	661	72.9
	Total	907	
Educational level	<High school	73	8.1
	High school	452	49.9
	University level degree	381	42.1
	Total	906	
Employment	Currently employed	409	45.2
	Currently unemployed or retired	52	5.8
	University student	444	49.0
	Total	905	

History for headache (“How often per month do you suffer because of headache”), car sickness (“How often do you suffer of car sickness when traveling by car”), and vertigo/dizziness (“How often per month do you suffer because of dizziness or vertigo”) were assessed with a 4 point Likert scale (never or almost never, sometimes, often, very often). Self perceived anxiety level (“would you define yourself as an anxious person?”) was assessed with a 5 point Likert scale (very often, often, sometime, almost never, never). The amount of time per day spent in front of a computer or a game console for work or leisure was assessed with a 4 point Likert scale (none, <1 hour, 1–4 hours, ≥ 5 hours).

#### 3D movie vision characteristics

We asked for the title of the last 3D movie seen, if the polarization glasses were used during the vision and when the movie was seen (< 1 month ago; 1–3 months ago; >3-6 months ago). An item regarded a health assessment on the week of the movie (“How was your health the week of the vision”) with 5 modalities (not remember, not good, fairly good, very good, excellent). Movie duration and type (animation movie or non animation movie) was inferred from the title using an online movie database (http://www.mymovies.it). Show time had 3 modalities (“movie starting time”, <20.30; 20.30-22.29, ≥22.30). We quantified enjoyment and appreciation of the movie (“Did you enjoy the movie?”) with a 5 point Likert scale (not at all, a little, moderately, a lot, extremely). This variable is possibly related to the level of attention given to the images during viewing that, in turn, may be correlated with the level of visual stress.

Additionally we asked two questions on the location of the sit in respect to the projection screen during the vision in the movie theatre. The first question refers to the proximity of the sit to the projection screen (near: sit within the first 3 rows from the projection screen; other: all the others). The second question refer to the angle of vision and choices were between lateral (last 2 sits in a row at right or left sides) or other positions (all the other sits in a row).

#### Choice of symptoms

Symptoms reported in current literature concerning visual stress from the use of head mounted displays, virtual reality, 3D displays and motion sickness (including simulator induced sickness) were compiled in a list and circulated among coauthors for final selection. Kennedy and colleagues in a recent paper[9] summarized VIMS symptoms into three general types of effects: (1) nausea; (2) oculomotor and (3) disorientation effects. Nausea relates to gastrointestinal distress such as nausea, stomach awareness, salivation, and burping. Oculomotor symptoms relates to visual fatigue symptoms such as tired or burning eyes, blurred or double vision, and headache. Disorientation relates to vestibular disturbance symptoms such as dizziness and vertigo[9]. We added palpitation as non specific symptom of autonomic nature because of the possible link with motion sickness [18].

We asked for presence of symptoms at 3 different times (during the 3D movie, just after the end and 2 hours later). The symptom section of the questionnaire has an internal consistency resulting in a Chronbach alpha of 0.66 [16].

#### Statistical analysis

We excluded from the analysis those individuals who did not remember the title of the movie seen, those who did not use the polarization glasses and those who did not report a good health condition during the week when they saw the movie.

Descriptive statistics were calculated to identify the frequency of selected individual and movie vision characteristics and symptoms.

Univariate analysis was used to evaluate the possible association between spectator and movie vision characteristics and VIMS symptoms estimating crude odds ratios (OR) with relative 95%CI and computing the Pearson Chi-square test. Fisher exact test probabilities were used when necessary.

For this analysis, the individual and movie vision characteristics were dichotomized as described below in order to run logistic models. Responses were coded as 1 for: often and very often headache, car sickness, vertigo/dizziness and anxiety; glasses or prescription lens use at cinema; >5 hours per day use of computer or game console; ≥ 22.30 movie starting time; no and little movie enjoyment; >100 minutes movie duration; animation movie. All other responses were coded as 0.

Symptom frequency was stratified by the time of on set (during the movie vision, right after, after 2 hours) and compared with a Chi square test. To increase the data consistency, for each symptom we pooled the “just after the movie” responses with the “after 2 hours” into the new variable “after the movie” responses (yes = 1; no = 0) when studying the univariate and multivariate associations of each symptom with responder characteristics. Statistical analysis of palpitation and double vision after the movie and of close and lateral position during vision were not carried out because of no or too low compliancy (<5% of individuals).

We estimated the recall bias on a small sub-sample of individuals (61) who were asked to compile the full questionnaire the day after the movie vision and the symptom section once again after 21 days from the movie vision. Consistency between the 2 measures was assessed with the Cronbach alpha statistics and were alpha = 0.89 for nausea symptoms, alpha = 0.90 for oculomotor symptoms and alpha = 0.78 for disorientation symptoms.

Adjusted logistic regression models were calculated for each visual symptom using age (modeled as continuous variable), gender (dichotomic) , educational level (dichotomized as university level = 1, all the others = 0) and employment status (dichotomized as university student = 1, all the others = 0) as covariates and one individual or movie vision characteristics per model. Additionally, we computed a series of logistic multivariate regression models including only those variables that had in the univariate analysis a Chi square p < 0.20 [19] using backward model selection.

All statistical analyses were carried out using the statistical software SPSS 19.0 (SPSS Inc., Chicago, USA).

## Results

### Characteristics of the study population

We collected 953 questionnaires. Of those, 46 individuals did not meet the inclusion criteria: 19 did not report the movie title or saw a 3D documentary, 27 defined their health in the week of the vision as not good. The final sample was composed by 907 individuals (attrition rate = 4.8%; Table 1).

Participants were between 18 and 65 years of age, with the category age 18–29 comprising 67.5% of responders, with slightly more females (54.0%) than males (46.0%). Most of responders were never married nor currently cohabitant with partner (72.9%), university students (49.0%) and with a high school level degree (49.9%).

Individual and movie vision characteristics are shown in Table 2. In our sample, 30.7% of individuals wore glasses and 12.7% prescription lenses when watching the 3D movie at cinema. In a typical month, 40.0% suffers headaches often or very often, 27.2% suffers often or very often of motion sickness when traveling by car, 22.7% suffers often or very often of dizziness/vertigo, 13.5% would often and very often define themselves as anxious person, 30.7% reports to use of computer and / or video game console for 5 hours or more per day.

**Table 2:** Individual and movie vision characteristics of study participants

**Variable**		**N**	**(%)**
Use of glasses or prescription lenses at cinema	No	514	56.7
	Yes, glasses	278	30.7
	Yes, lens	115	12.7
	Total	907	
Headache	Never or almost never	158	17.4
	Sometimes	386	42.6
	Often	257	28.3
	Very often	106	11.7
	Total	907	
Car sickness	Never or almost never	330	36.4
	Sometimes	330	36.4
	Often	143	15.8
	Very often	104	11.5
	Total	907	
Vertigo	Never or almost never	396	43.7
	Sometimes	305	33.6
	Often	174	19.2
	Very often	32	3.5
	Total	907	
Anxiety	Never or almost never	339	45.3
	Sometimes	287	38.4
	Often	86	11.5
	Very often	36	4.8
	Total	748	
Daily use of computer and/or video game console	None	28	3.1
	<1 hour	157	17.3
	1-5 hors	444	49.0
	>5 hours	278	30.7
	Total	907	
Position during vision: proximity to the screen	Sit within first 3 raw from screen	72	7.9
	Other	835	92.1
	Total	907	
Position during vision: proximiy to the screen and lateral view	Sit within first 3 raws and lateral	20	2.2
	Other	887	97.8
	Total	907	
Showtime (movie starting time)	Before 20.30	131	17.4
	20.30-22.29	343	45.5
	22.30 or later	280	37.1
	Total	754	
Enjoiment of the movie	Not enjoyable	25	3.3
	A little	56	7.4
	Moderately	148	19.6
	Very	369	48.9
	Extremely enjoyable	156	20.7
	Total	754	
Movie duration	<= 100 minutes	347	38.3
	>100 minutes	560	61.7
	Total	907	
Movie type	Animated film	572	63.1
	Non Animated film	335	36.9
	Total	907	

At the day of questionnaire administration, almost half of responders (46.0%) reported to have seen a 3D movie in the previous 4 weeks (70.6% in the previous 3 months). Movie titles indicated by responders included Avatar, Despicable me, Ice age 3: dawn of dinosaurs, Megamind, Saw VI, Tangled, The Chronicles of Narnia: the voyage of the dawn treader, Tron legacy, Up, Wonderland Alice. Animation movies were 63.1% of total and movie duration was comprised between 82 and 162 minutes. Most of the responders saw an evening movie starting at 20.30 or later (82.6%), few individuals (7.9%) were sitting in the cinema at the first 3 front rows and even fewer at front and lateral position (2.2%) during the movie vision. Appreciation of the movie was high (a lot or extremely enjoyable) in 69.6% of individuals.

### Frequency and duration of symptoms

**Figure 1:**
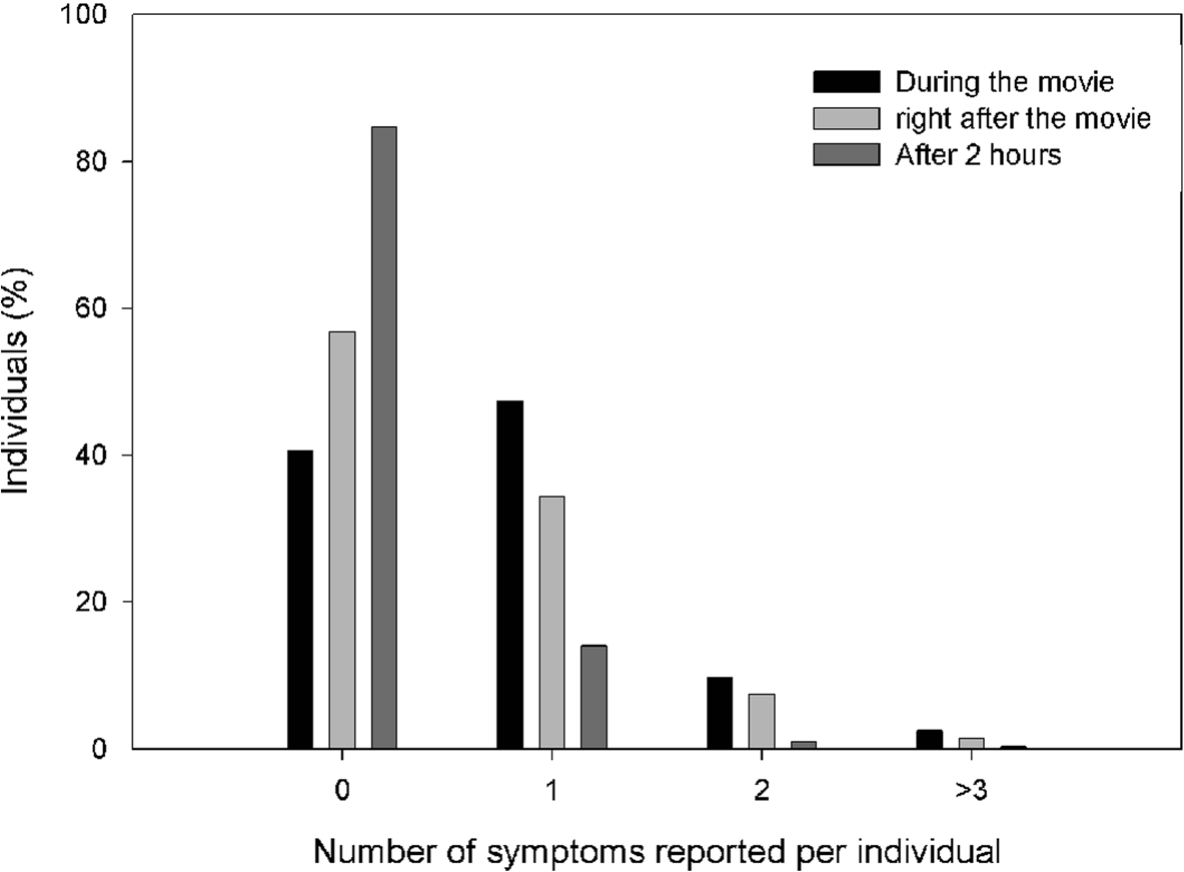
**Symptoms reported per individual after viewing a 3D movie.** Symptoms were reported at 3 different times (during, right after and after 2 hours from the movie vision).

In our sample, at least one symptom was reported by 602 (66.4%) individuals. Of these, 539 (60.4%) individuals reported 1 or more symptoms during the movie, 392 (43.2%) right after and 139 (15.3%) at 2 hours from vision (Figure 1). The number of symptoms reported per individual diminished with the time from vision (Table 2; r x c test, 1 symptom per individual: Pearson Chi Square = 235.3, p < 0.001; 2 symptoms per individual: Pearson Chi Square = 64.4, p < 0.001; 3 or more symptoms per individual: Pearson Chi Square = 15.6, p < 0.001).

**Figure 2:**
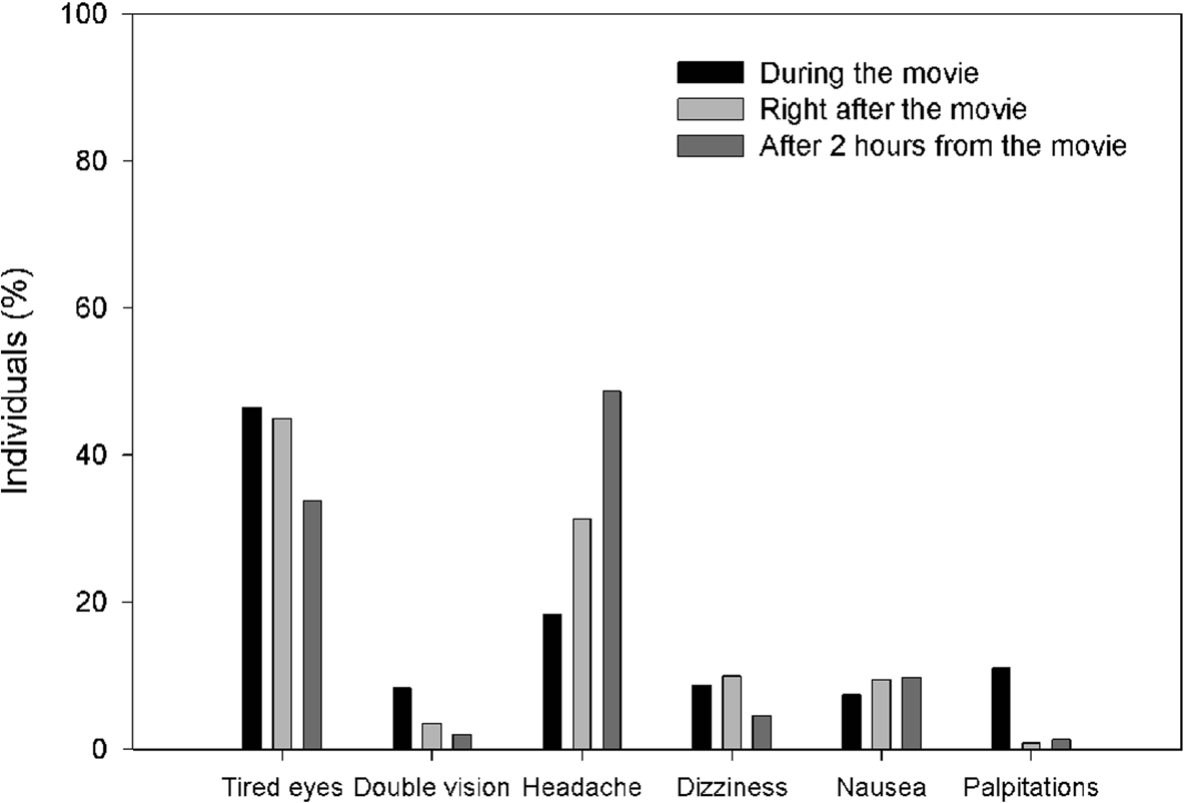
**Number of spectators of a 3D movie reporting symptoms.** Symptoms were reported at 3 different times (during, right after and after 2 hours from the movie vision). The same individual could report more than one symptom if necessary.

The most frequent symptoms reported during the movie were tired eyes (34.8%), headache (13.7%) and palpitations (8.3%, Figure 2). Tired eyes and headache were also the most frequently reported symptoms right after the movie (respectively by 24.0% and 16.8% of individuals) and after 2 hours from the movie (respectively by 5.7% and 8.3% of individuals).

Frequency of individuals reporting symptoms decreased with time from the vision (r x c test, tired eyes: Pearson Chi Square = 232.4, p < 0.001; double vision: Pearson Chi Square = 61.3, p < 0.001; headache: Pearson Chi Square =29.8, p < 0.001; dizziness: Pearson Chi Square = 41.2, p < 0.001; nausea: Pearson Chi Square = 20.7, p < 0.001; palpitations: Pearson Chi Square = 132.0, p < 0.001).

The vanishing time was different for each symptom. Double vision and palpitations were barely reported just after the movie (by <2% of individuals) and were almost absent at 2 hours from the vision. Dizziness and nausea were still present just after the movie (respectively in 5.3% and 5.1% of individuals) and barely reported at 2 hours (by <2% of individuals) when only tired eyes and headache were still reported with frequencies > 5%.

In general, among those individuals reporting symptoms after the movie, a large proportion of them already reported the same type of disturb during the vision of the movie. For example, out of 218 individuals reporting tired eyes right after the movie, 155 (71.1%) already suffered of the same symptom during the movie. Similarly, out of 52 individuals reporting tired eyes after 2 hours, 46 (88.5%) already suffered of the same symptom during the movie.

### Association with individual and movie vision characteristics

During and after the movie, many of the visually induced symptoms tested in this study were associated with individual and / or movie vision characteristics. Crude and adjusted OR (95% CI) are shown in Table 3 and 4.

**Table 3:** Crude and adjusted OR (95% CI) of selected spectator and movie vision characteristics and visually induced symptoms during the vision of a 3D movie. Adjusted for age, gender, educational level, employment status and movie appreciation

		**Tired eyes**	**Double vision**	**Headache**	**Dizziness**	**Nausea**	**Palpitations**
Use of glasses or prescription lenses	Crude OR Adjusted OR	0.94(0.70-1.26) 0.96(0.68-1.35)	0.53 (0.27-1.05)° 0.54(0.27-1.08)°	1.19 (0.79-1.77) 1.22 (0.81-1.84)	1.60 (0.93-2.75)° 1.63(0.94-2.81)°	0.55 (0.27-1.11)° 0.55(0.27-1.13)°	1.48(0.91-2.40)§ 1.33(0.80-2.22)
Headache often and very often	Crude OR Adjusted OR	1.34 (1.01-1.77)* 1.36(1.02-1.80)*	1.94 (1.12-3.34)* 1.96 (1.13-3.40)*	2.17(1.48-3.18)** 2.09(1.42-3.09)**	1.09 (0.61-1.94) 1.13 (0.67-1.95)	1.09 (0.61-1.94) 0.92 (0.50-1.67)	2.92(1.78-4.77)** 3.26(1.95-5.44)**
Carsickness often and very often	Crude OR Adjusted OR	1.13(0.83-1.53) 1.20 (0.88-1.65)	1.40 (0.79-2.49) 1.42 (0.79-2.55)	1.44 (0.96-2.16)° 1.39 (0.91-2.11)§	2.23(1.31-3.82)** 2.16(1.24-3.77)**	3.12(1.75-5.54)** 3.07(1.68-5.60)**	9.17(5.4-15.68)** 11.0(6.17-19.70)**
Recurrent vertigo >1 episode per month	Crude OR Adjusted OR	0.98 (0.71-1.36) 1.00(0.72-1.39)	1.67 (0.93-2.99)° 1.68(0.93-3.03)°	1.77(1.17-2.68)** 1.71(1.13-2.63)*	2.32(1.34-4.02)** 2.19(1.26-3.81)**	1.21 (0.63-2.32) 1.11(0.57-2.14)	8.67 (5.20-14.44)** 0.20(5.95-17.70)**
Use of computer or videogame console > 5 h per day	Crude OR Adjusted OR	1.20 (0.90-1.61) 1.19 (0.88-1.65)	1.08 (0.60-1.92) 1.21 (0.66-2.25)	0.80 (0.52-1.22) 0.86 (0.54-1.35)	1.17 (0.67-2.05) 1.03 (0.56-1.87)	1.17 (0.64-2.15) 1.26 (0.65-2.42)	1.14(0.69-1.89) 1.12(0.65-1.93)
Self perceived high anxiety level	Crude OR Adjusted OR	1.58 (1.08-2.33)* 1.53 (1.02-2.28)*	1.84 (0.94-3.59) 1.61 (0.81-3.22)§	1.90(1.17-3.07)** 1.61 (0.98-2.66)°	1.17 (0.56-2.45) 1.01 (0.48-2.15)	1.44 (0.68-3.05) 1.16 (0.53-2.52)	-
Showtime (movie starting time ≥22.30)	Crude OR Adjusted OR	1.18 (0.88-1.59) 1.01 (0.74-1.37)	0.89 (0.49-1.62) 0.76 (0.41-1.41)	1.62 (1.10-2.40)* 1.44 (0.95-2.18)°	0.55 (0.29-1.06)§ 0.48 (0.24-0.97)*	0.69 (0.36-1.40) 0.56 (0.27-1.15)§	-
Movie type: animation movie	Crude OR Adjusted OR	0.58(0.44-0.77)** 0.64(0.48-0.86)**	0.71 (0.41-1.22) 0.75 (0.43-1.30)	0.81 (0.55-1.20) 0.91 (0.60-1.36)	2.17 (1.15-4.07)* 2.51 (1.29-4.90)*	1.04 (0.58-1.89) 1.22 (0.64-2.30)	5.42(2.57-11.44)** 4.35(2.03-9.30)**
Position during vision close to the screen	Crude OR Adjusted OR	0.87 (0.52-1.45) 0.81 (0.48-1.37)	0.41(0.10-1.73) 0.38 (0.09-1.62)	1.02 (0.51-2.05) 0.96 (0.47-1.96)	1.92 (0.87-4.22) 1.90 (0.85-4.24)§	1.31 (0.50-3.41) 1.24 (0.46-3.29)	2.45(1.25-4.79)* 3.08(1.52-6.21)**
Movie duration >100 min	Crude OR Adjusted OR	1.11 (0.83-1.47) 1.10 (0.82-1.47)	1.33 (0.75-2.37) 1.46 (0.81-2.63)	0.89 (0.59-1.28) 0.88 (0.59-1.32)	1.22 (0.70-2.13) 1.19 (0.67-2.11)	0.92 (0.52-1.66) 0.89 (0.49-1.63)	2.43(1.38-4.31)** 2.24(1.25-4.00)*

** p < 0.01;* p <0.05; ° p < 0.1; § p < 0.2; -: non computable.

**Table 4:** Crude and adjusted OR (95% CI) of selected spectator and movie vision characteristics and visually induced symptoms after the vision of a 3D movie. Adjusted for age, gender, educational level, employment status and movie appreciation

		**Tired eyes**	**Headache**	**Dizziness**	**Nausea**
Use of glasses or prescription lenses	Crude OR Adjusted OR	0.75(0.54-1.06) § 0.78(0.55-1.10) §	1.06 (0.74-1.51) 1.08(0.75-1.55)	1.66 (0.92-3.01)° 1.67(0.92-3.06)§	1.21 (0.66-2.23) 1.25(0.67-2.32)
Headache often and very often	Crude OR Adjusted OR	1.12 (0.82-1.52) 1.10(0.81-1.51)	1.80 (1.29-2.52)** 1.81(1.29-2.54)**	1.28 (0.72-2.30)§ 1.07(0.59-1.95)	1.60 (0.90-2.85)§ 1.39(0.78-2.52)
Carsickness often and very often	Crude OR Adjusted OR	0.66 (0.46-01.01) 0.67(0.46-0.97)*	1.54 (1.08-2.19)* 1.57(1.09-2.26)*	1.99 (1.10-3.60)* 1.72(0.93-3.16)°	2.73 (1.53-4.88)** 2.82(1.54-5.18)**
Recurrent vertigo >1 episode per month	Crude OR Adjusted OR	1.28 (0.84-1.97) 1.17(0.75-1.82)	1.94 (1.26-3.01)* 1.73(1.10-2.71)*	3.30 (1.73-6.28)** 2.72(1.39-5.34)**	1.76 (0.85-3.63)§ 1.68(0.79-3.58)§
Use of computer or videogame console > 5 h per day	Crude OR Adjusted OR	0.92 (0.66-1.28) 0.86(0.61-1.22)	0.99 (0.69-1.42) 1.02(0.69-1.49)	0.93 (0.49-1.76) 0.90(0.45-1.79)	1.33 (0.73-2.43) 1.17(0.61-2.24)
Self perceived high anxiety level	Crude OR Adjusted OR	1.69 (1.12-2.54)* 1.58(1.04-2.40)*	2.16 (1.41-3.30)** 1.96(1.26-3.04)**	1.03 (0.60-2.86) 1.02(0.46-2.29)	0.89 (0.37-2.14) 0.83(0.34-2.04)
Showtime (movie starting time ≥22.30)	Crude OR Adjusted OR	1.16(0.84-1.60) 1.04(0.75-1.46)	1.63(1.15-2.29)* 1.43(0.99-2.04)°	0.82(0.43-1.58) 0.78(0.39-1.56)	0.63(0.32-1.26)§ 0.62(0.30-1.29)
Movie type: animation movie	Crude OR Adjusted OR	0.80 (0.59-1.09)§ 0.86(0.62-1.18)	0.68 (0.49-0.96)* 0.75(0.53-1.1)°	1.18(0.64-2.19) 1.22(0.64-2.32)	0.84 (0.47-1.51) 0.81(0.44-1.51)
Position during vision: close to the screen	Crude OR Adjusted OR	1.25 (0.73-2.13) 1.20(0.70-2.05)	1.12 (0.62-2.03) 1.06(0.58-1.94)	0.76(0.23-2.52) 0.76(0.23-2.55)	0.75 (0.23-2.46) 0.75(0.23-2.51)
Movie duration >100 min	Crude OR Adjusted OR	0.91 (0.67-1.24) 0.94(0.69-1.29)	0.90 (0.64-1.26) 0.92(0.65-1.31)	1.03 (0.57-1.89) 1.06(0.57-1.97)	1.18 (0.64-2.15) 1.09(0.59-2.03)

** p < 0.01;* p <0.05; ° p < 0.1; § p < 0.2.

Briefly, suffering of headache often and very often during a typical month was associated with the increased probability of suffering tired eyes (adjusted OR = 1.36; p < 0.05), double vision (adjusted OR = 1.96; p < 0.05), headache (adjusted OR = 2.09; p < 0.01) and palpitations (adjusted OR = 3.26; p < 0.01) during the movie and with headache (adjusted OR = 1.81; p < 0.01) after the movie. Sensitivity to car sickness was associated with increased probability of dizziness (adjusted OR = 2.16; p < 0.01), nausea (adjusted OR = 3.07; p < 0.01) and palpitations (adjusted OR = 11.0; p < 0.01) during the movie and with tired eyes (0.67; p < 0.05) headache (adjusted OR = 1.57; p < 0.05) and nausea (adjusted OR = 2.82; p < 0.01) after the movie. Sensitivity to dizziness/vertigo was associated with increased probability of headache (adjusted OR = 1.71; p < 0.05), dizziness (adjusted OR = 2.19; p < 0.01), and palpitations (adjusted OR = 10.20; p < 0.01) during the movie and with headache (adjusted OR = 1.73; p < 0.05) and dizziness (adjusted OR = 2.72; p < 0.01) after the movie. Self perceived high anxiety level was associated both during and after the movie with an increased probability of tired eyes (during: adjusted OR = 1.53; after: adjusted OR = 1.58, all p < 0.05) and after with headache (adjusted OR = 1.99; p < 0.05). Late showtime was associated with decreased probability of dizziness after the movie (adjusted OR = 0.48; p < 0.05). The vision of animation movies decreased the probability of tired eyes (adjusted OR = 0.64; p < 0.01), but increased the probability of dizziness (adjusted OR = 2.51; p < 0.05) after the movie. The position close to the screen and the movie duration >100 minutes were associated with increased probability of palpitations during the movie (respectively adjusted OR = 3.08; p < 0.05).

Use of prescription glasses or lens and daily use of computer and/or videogame console for more than 5 hours were not associated with any symptom reported during and after the movie.

### Logistic models

The best models predicting VIMS symptoms from individual and movie vision characteristics are shown in Table 5. For each symptom, predictors were selected with a backward elimination procedure adjusted for age, gender, educational level (coded as bivariate: university level degree versus other), employment status (coded as bivariate: student versus other) and appreciation of the movie (coded as bivariate: low enjoyment versus other) starting from the pools of variables resulting associated with p < 0.2 at adjusted analysis. Best predictors for increased probability of tired eyes during the 3D movie were frequent headache (OR = 1.34; p < 0.05) while viewing an animation movie was protective (OR = 0.65; p < 0.05). After the movie best predictors of tired eyes were high anxiety level (OR = 1.58; p < 0.05) while frequent car sickness was protective (OR = 0.67; p < 0.05). Double vision during the movie was predicted by frequent headache (OR = 1.96; p < 0.05). Headache during the movie was predicted by frequent headache (OR = 2.09; p < 0.05) while after the movie by frequent car sickness (OR = 1.52; p < 0.05), high anxiety (OR = 1.88; p < 0.05), frequent headache (OR = 1.64; p < 0.05). Dizziness during the movie was predicted by sensitivity to dizziness (OR = 1.81; p < 0.05) and animation movie (OR = 2.32; p < 0.05) and by dizziness (OR = 2.38; p < 0.05) after the movie. Nausea during the movie was predicted by frequent car sickness during (OR = 3.07; p < 0.01) and after the movie (OR = 2.83; p < 0.01). Finally palpitations during the movie were predicted by frequent car sickness (OR = 5.93; p < 0.01) and dizziness (OR = 5.45; p < 0.01).

**Table 5:** Logistic regression models selected with backward elimination procedure predicting symptoms during and after the movie vision

**Symptom**	**Predictor variables**	
	**During the movie**	**After the movie**
Tired eyes	Head ache often and very often 1.34 (1.01-1.79) Animation movie 0.65(0.48-0.87)	Car sickness often and very often 0.67 (0.47-0.97) Self perceived high anxiety level 1.58 (1.03-2.40)
Double vision	Headache often and very often 1.96 (1.13-3.41)	-
Headache	Headache often and very often 2.09 (1.41-3.10)	Car sickness often and very often 1.52 (1.04-2.22) Self perceived high anxiety level 1.88 (1.20-2.94) Headache often and very often 1.64 (1.16-2.32) Showtime late 1.49 (1.03-2.16)
Dizziness	Dizziness often and very often 1.81 (1.00-3.27) Animation movie 2.32 (1.17-4.57)	Dizziness often and very often 2.38 (1.30-4.35)
Nausea	Car sickness often and very often 3.07(1.69-5.60)	Car sickness often and very often 2.83 (1.54-5.18)
Palpitations	Car sickness often and very often 5.93(3.21-10.97) Dizziness often and very often 5.45(3.03-9.79)	-

All models are adjusted for age, gender, educational level, employment status and movie appreciation. For each symptom, the OR and 95%IC of significant predictor variables (p < 0.05) are shown. Footnotes: - not computable because of low compliancy (<5%).

## Discussion

The most striking result of our analysis is the high occurrence of symptoms reported by spectators during and after the vision of a 3D movie in a cinema. Two thirds of individuals reported 1 or more symptoms during the movie, more than one third right after the movie and one quarter still after 2 hours. However, it should be remarked that most of the individuals reported mild symptoms that are barely clinically relevant and that disappeared quickly.

The frequency of spectators reporting symptoms in our study is roughly similar to those observed by others in controlled experimental settings on a smaller number of participants [15], [8] and support their findings. Polonen and colleagues[15] used the simulator sickness questionnaire [20] and a visual strain questionnaire to quantify the level of visual comfort of spectators before and after the movie “U2 3-D”, one of the first filmed with the 2 parallel digital camera system. In their study (sample size = 84 people), 50% of the people had some sickness symptoms after the movie, but in general, symptom severity was relatively low and often related to visual discomfort. Only two individuals experienced strong signs of visually induced motion sickness and 4 other people had a large increase in symptom severity after the movie [15].

In our study, we did not ask to participants to rate the intensity of the symptoms suffered but it is reasonable to think that individuals still reporting symptoms after 2 hours (25.3% in our study) were those with most severe sickness.

Most symptoms experienced by 3D movie spectators disappeared as soon as the movie ended (Figure 2). The rapid dissipation of symptoms agrees with previous findings on the effects of head mounted displays, that disappeared within 10 min from the end of their use [14].

Although the low number of cases reported at 2 hours hindered a proper analysis of association with characteristics of individuals, it seems that some people recover slower than other after the intense visual stimulations experienced during a 3D movie. Research should be directed towards identify the reasons of this behavior.

The highest frequency of symptoms in our study refers to visual fatigue (tired eyes, double vision, headache). This result resembles earlier observation that the prolonged use of 3D displays produces visual fatigue and discomfort in the viewers [21], [22], [23]. A possible explanation is connected to the vergence-accomodation conflict [24]. The causal mechanism relays on the fact that the eye focus mechanisms (accomodation and blur in the retinal image) specify the depth of the display rather than the depth of the movie scene. Thus the uncoupling of vergence and accommodation required by 3D displays may reduce the ability to fuse the binocular stimulus and causes discomfort and fatigue in the viewers [24]. For example, Emoto and collegaues [21] reported symptoms of visual fatigue when the observers viewed stimuli with a larger conflict between the vergence and the focal distances. Kuze and Ukai [8] showed a significant increase of individuals reporting eyestrain, general discomfort and focusing difficulty when viewing a stereoscopic movie compared to a conventional 2D movies. This might also relate to a more intense eye movements in viewers of 3D movies compared to 2D movies [25]. In their recent study Hakkinen and colleagues found that in a 2D movie viewers tend to focus at the actors while the eye movement patterns of 3D viewers were more widely distributed to other targets such as complex stereoscopic structures and objects nearer than the actors [25]. This behavior might increase the vergence-accomodation mismatch, increasing the visual stress on 3D spectators.

Besides visual fatigue symptoms, a portion of viewers reported dizziness and/or nausea. Nausea, dizziness and vertigo relate to vestibular disturbance and may be due to a mismatch between the sensed and the expected vertical position of the body that have been recently suggested as responsible mechanism for causing VIMS [11]. In Japan Ujike [26] studied the Matsue movie sickness incident, when 36 out of 294 junior high school students were treated at the hospital for symptoms of motion sickness after watching a hand camera made movie characterized by unexpected whole image motion and vibration. Interestingly, the students who reported greater severity were distributed around the front row and center positions, and those who reported less severity were distributed around the backmost row. For the authors this may indicate that the critical visual angle, above which the risk of visually induced motion sickness noticeably increase, may be between the visual angles obtained in the front row and at the end of the row, at least in the conditions of the incident [26].

Regarding the associations between symptom occurrence and individual characteristics, we found a significant association between the number of individuals reporting visually induced symptoms during the movie and history of headache, car sickness, dizziness/vertigo but not with the use of computer and/or videogame console for more than 5 hours per day. The above mentioned associations were not surprising as the relationships between motion sickness, vertigo, dizziness and migraine is well documented [27], [28], [29]. Also the association between high level of (perceived) anxiety and headache after the movie is probably connected to individual stress or tension, a typical trigger of headache [30].

Symptoms like tired eyes and double vision could be associated to anxiety because of the worsening of a pre-existing physiological condition, as can occur in a psychosomatic process.

Among movie vision characteristics, low enjoyment of the movie was associated with several visually induced symptoms. However, it is not possible to infer if the low enjoyment was the result of the symptom onset or a predictor of them. Animation 3D movies seems to be protective for tired eyes but not for disorientation type symptoms. Notably showtime, sitting close to the screen during projection and movie duration all had little and not significant effects. The only exception was palpitation during the movie that resulted associated with several individual and movie vision characteristics. However, the large confidence intervals of OR resulting from low reporting in several factor per symptom combinations hindered a proper interpretation of those results.

This study has also several limitations that are relevant for a thorough evaluation of its findings. Most notably, all results are based on data collected through a self-administered questionnaire to not primed individuals. Some people might have been not familiar with the descriptions of symptoms in the questionnaire, although we did not choose to use more complex and long instruments (like the SSQ [20]) to avoid such problem. It is also possible that only people who remembered to have suffered of some health problem during or after the 3D movie actually agreed in answering the questionnaire while those who did not have problems, did not. The lack of a control group is also a limitation of this study as some individuals might be sensitive to triggers of symptoms beyond the 3D movie vision. For example, visual stimuli like flickering lights, striped patterns, TV and 2D movie could also act as triggers of migraine [17] or videoterminal prolonged use may cause visual fatigue [10]. However, given the cross sectional nature of this research, we did not have among the aims the investigation of possible causal relationships and a control group was not strictly necessary. Additionally, although we posed as inclusion criteria to have seen the 3D movie within 6 months from questionnaire administration, and a large portion of participants (N = 417; 46%) declared to have seen a 3D movie within a month from the questionnaire administration, some answers might be affected by recall bias. Nevertheless, the assessment of recall bias on a subsample of individuals gave satisfactory results indicating that the bias should be minimal at least for those that administered the questionnaire within 3 weeks from the day of the movie.

## Conclusion

The high occurrence of visually induced symptoms resulting from this survey along with the increasing commercial releases of 3D movies suggest the need of informing the public on some discomfort they might suffer during and after the 3D movie. Those individuals who report more severe symptoms might have some disorder of their binocular vision and an optometrist examination could be advisable.

Our study, conducted in real life conditions, revealed some factors associated with visually induced symptoms that were not fully assessed in previous experimental experiences. Real life conditions add components such as the length of the movie, the position of the sit in respect to the screen (e.g. the angle of view and the proximity to the screen of the viewer) and permit a more general assessment of the inter individual differences in susceptibility, that are potential predictor of visually induced symptoms. Future research should address age groups not yet considered in previous works, such as children and young adults. Analytic and experimental studies should be conducted to gain a robust understanding of the causes of VIMS symptoms.

## Competing interests

The authors do not have any financial or non-financial competing interests.

## Authors' contributions

AGS and GLT conceived the study and participated in the design and coordination. All authors participated in the data collection. AGS and AM made the statistical analysis. All authors participated in the interpretation of results. AGS made the first draft of the manuscript with contributions from DDT. GLT critically revisited the manuscript. “All authors read and approved the final version of the manuscript.”

## Pre-publication history

The pre-publication history for this paper can be accessed here:

http://www.biomedcentral.com/1471-2458/12/779/prepub
